# Bone Marrow Mesenchymal Stem Cell-Derived Extracellular Vesicles Carrying circ_0050205 Attenuate Intervertebral Disc Degeneration

**DOI:** 10.1155/2022/8983667

**Published:** 2022-07-05

**Authors:** Xiao-Jun Yu, Qi-Kun Liu, Rui Lu, Shan-Xi Wang, Hao-Ran Xu, Ying-Guang Wang, Yuan Bao, Yong-Qiao Jiang, Meng-Wei Li, Hao Kang

**Affiliations:** Department of Orthopedics, Tongji Hospital, Tongji Medical College, Huazhong University of Science and Technology, Wuhan 430030, China

## Abstract

**Objective:**

It has been reported that bone marrow mesenchymal stem cells (BMSCs) are a potential source of autologous stem cells to support the nucleus pulposus (NP) regeneration in intervertebral disc degeneration (IDD). Herein, we aim to study the mechanism underlying the effects of BMSC-derived extracellular vesicles (BMSC-EVs) on nucleus pulposus cells (NPCs) in IDD.

**Methods:**

EVs were isolated from BMSCs. An IDD model was surgically established in C57BL/6J mice. NPCs were exposed to tBHP to establish an IDD cell model. RNA sequencing was performed to identify differentially expressed circRNAs in NP tissues harvested from mice with IDD. Interactions among circ_0050205, miR-665, and GPX4 were validated, and different interventions were used to study the roles of these molecules in NPC biological functions.

**Results:**

BMSC-EVs promoted NPC survival and inhibited NPC apoptosis and extracellular matrix (ECM) degradation. circ_0050205 expression was downregulated in the NP tissues of IDD mice, and BMSC-EVs facilitated NPC survival and suppressed ECM degradation in NPCs by transferring circ_0050205. circ_0050205 sponged miR-665 and upregulated GPX4 expression. BMSC-EVs expressing circ_0050205 promoted NPC survival-inhibited ECM degradation in NPCs and alleviated IDD in mice via the miR-665/GPX4 axis.

**Conclusion:**

In conclusion, BMSC-EVs promoted NPC survival-inhibited ECM degradation in NPCs and attenuated IDD progression via the circ_0050205/miR-665/GPX4 axis.

## 1. Introduction

Intervertebral disc degeneration (IDD) is considered a major contributor to lower back pain, and even disability, worldwide [1]. IDD results from excessive destruction of the extracellular matrix (ECM) due to an imbalance between catabolism and anabolism in discs, and IDD represents an abnormal and cell-mediated response to progressive structural failure [2, 3]. Nucleus pulposus cells (NPCs), the primary cell type constituting the nucleus pulposus (NP), bear great responsibility in the generation of extracellular matrix (ECM) molecules [4]. Importantly, NPC dysfunction and ECM degradation are the major causes of IDD [5, 6]. Furthermore, oxidative stress and ferroptosis in the NP play crucial roles in the pathogenesis of IDD [7, 8].

Recently, cell-based strategies have been considered in attempts to regenerate NP tissues to affect IDD [9]. Specifically, bone marrow mesenchymal stem cells (BMSCs) have been applied in rabbit and pig models of IDD as BMSC transplantation has the ability to facilitate IVD regeneration [10, 11]. It has been reported that MSCs possess their therapeutic potentials by paracrine signaling through releasing bioactive factors and soluble peptides [12]. Extracellular vesicles (EVs), nanosized vesicles (30 to 100 nm in diameter), are produced by the MSCs and act importantly in cell-cell communication by carrying various biomolecules, including DNAs, lipids, proteins, and noncoding RNAs; EVs have the ability to drive regenerative processes in many diseases [13].

Circular RNAs (circRNAs), a newly identified group of endogenous noncoding RNAs, are involved in NPC functions and ECM synthesis or degradation [14]. Interestingly, circRNAs have been shown to be enriched and expressed at stable levels in EVs [15], and thus, circRNAs mediate the effects of EVs on IDD [16]. There is increasing interest in investigations of a new paradigm for understanding the regulatory mechanisms of ncRNAs, including circRNAs, microRNAs (miRNAs), and lncRNAs, termed the competitive endogenous RNA (ceRNA) hypothesis. The circRNA acts as ceRNAs or natural miRNA sponges and therefore results in increased expression of the corresponding miR target genes [14, 17]. Based on the aforementioned evidence, we are interested in further understanding the circRNA-based mechanism underlying the paracrine effects of BMSCs on NPCs. In this study, we first performed RNA sequencing to identify differentially expressed circRNAs in the NP tissues harvested from the established IDD mouse models. EVs were then isolated from mouse BMSCs (mBMSCs) and cocultured with the NPCs exposed to tBHP to evaluate the effect of BMSC-derived EVs (BMSC-EVs) on the NPC biological functions. Next, we investigated the transfer of a candidate circRNA from BMSCs to NPCs via EVs and further constructed a ceRNA regulatory network related to the function of this candidate circRNA in IDD. This study will reveal the molecular mechanism of IDD and offers a new target for the treatment of IDD.

## 2. Materials and Methods

### 2.1. Ethics Statement

Animal experiments were ratified by the animal ethics committee of Tongji Hospital, Tongji Medical College of Huazhong University of Science and Technology.

### 2.2. IDD Mouse Model Establishment

A total of 40 C57BL/6J male mice (age: 12 weeks, weight: 18–22 g) were procured from the Beijing Institute of Pharmacology and Toxicology, Chinese Academy of Medical Sciences (Beijing, China). All mice were housed in a specific pathogen-free-grade environment with conditions of 60–65% humidity, at 22–25°C under a12 h light/dark cycle (eat and drink freely). After 7 days of acclimation, the IDD model was prepared in C57BL/6J mice by annulus fibrosis (AF) needle puncture as previously reported [18]. First, the mice were administered with ketamine (100 mg/kg) for general anesthesia. A sagittal small skin incision was made from the Co6 to Co8 coccygeal discs to locate the disc position to insert the needles in the tail. Next, a puncture was made vertically with a syringe needle at the Co6-Co7 disc, followed by 180° axial rotation; then, the needle position was maintained for 10 s. A puncture was made parallel to the endplates with a 31-G needle through the AF into the nucleus pulposus, and the needle was inserted 1.5 mm into the disc to depressurize the nucleus. Other undisturbed segments were considered controls. Six weeks after surgery, the Co6-Co7 disc was harvested for further investigation.

The mice were randomized into a sham group (mice received sham operation), aIDD group (mice received IDD model establishment), a BMSC-EV + vector group (IDD mice injected with EVs from vector-transduced BMSCs), a BMSC-EV + LV-circ_0050205 + sh-NC group (IDD mice injected with EVs from BMSCs transduced with adenovirus carrying LV-circ_0050205 + sh-NC), and a BMSC-EV + LV-circ_0050205 + sh-GPX4 group (IDD mice injected with EVs from BMSCs transduced with adenovirus carrying LV-circ_0050205 + sh-GPX4) (8 mice in each group). Adenovirus (10 *μ*L, 5 × 10^9^ PFU, Sangon Biotech, Shanghai, China) was immediately injected into the puncture site. A week later, the mice (except the sham-operated mice) were injected with EVs (3 *μ*g/*μ*L in 100 *μ*L) via the caudal vein once a week for 6 consecutive weeks. Finally, the mice were euthanized and the NP tissues were harvested and isolated for further experiments.

### 2.3. Radiographic Analysis

Sedated mice underwent X-ray after IVD puncture and treatment. X-ray was started in a 21 lp/mm detector which provided over 5x geometric magnification (Faxitron VersaVision; Faxitron Bioptics LLC, Tucson, AZ. USA). The disc height indices (DHIs) were calculated by three spinal surgeons who were blinded to the treatment utilizing the following formula: DHI = IVD height/adjacent IVD body height [19].

### 2.4. Histological Examinations

NP tissues were rinsed in normal saline, fixed with 4% paraformaldehyde (30–50 min), dehydrated, cleared, paraffin embedded, and sliced. The slices were mounted onto slides and then heated at 45°C. For hematoxylin-eosin (HE) staining, after deparaffinization and dehydration with gradient alcohol, the slides were stained with hematoxylin for 5 min. Then, 1% hydrochloric acid ethanol was added and incubated to differentiate the slices for 3 s. The slices were then incubated in 5% eosin for 3 min, dehydrated with gradient ethanol, and cleared in xylene. For oil red O staining, after deparaffinization and dehydration with gradient alcohol, the slides were stained with hematoxylin for 5 min and hydrolyzed in 1% hydrochloric acid ethanol for 15 s. The slices were stained with aqueous solutions of 0.02% malachite green for 3 min and rapidly washed with 1% ethylic acid, followed by staining with 0.1% oil red O for 3 min. The sections were dehydrated with gradient alcohol and permeabilized with xylene. Finally, the slides were observed under an optical microscope and 10 random views were selected per slide.

### 2.5. Immunohistochemistry

Paraffin-embedded NP tissue slices (4 *μ*m thickness) were placed in ethylenediaminetetraacetic acid (EDTA) buffer solution (0.05 mol/L Tris + 0.001 mol/L EDTA; pH 8.5) and heated for 21 min in a microwave oven for antigen retrieval. The tissue slices were incubated in 0.3% H_2_O_2_ for 10 min, followed by blocking using 5% bovine serum albumin (BSA) for 20 min. The tissue slices were probed overnight at 4°C with the following primary antibodies: rabbit anti-MMP13 (ab219620, 1: 500, Abcam, Cambridge, UK), rabbit anti-ADAMTS 5 (PA5-27165, 1 : 100, Invitrogen, Carlsbad, CA), rabbit anti-COL II (15943-1-AP, 1 : 200, Proteintech Group Inc., Chicago, IL), rabbit anti-aggrecan (13880-1-AP, 1 : 200, Proteintech), and rabbit anti-GPX4 (ab125066, 1 : 100, Abcam). Then, the slices were further incubated with biotinylated secondary antibody goat anti-rabbit IgG (ab205718, 1 : 2500, Abcam) for 20 min. The slices were washed with 0.1 M PBS and reprobed with streptomycin albumin working solution labeled with horseradish peroxidase for 20 min. Next, the slices were colored with diaminobenzidine, counterstained with hematoxylin, and observed under a microscope (Leica-DM2500, Leica, Wetzlar, Germany). Staining quantification was performed using ImagePro Plus 7.1 software (Media Cybernetics, Silver Spring, MD).

### 2.6. RNA Extraction and Sequencing

Total RNA was extracted from NP tissues of mice using TRIzol reagent (Invitrogen), and the RNA concentration was measured at wavelengths of 260/280 utilizing a NanoDrop ND-1000 spectrophotometer (Thermo Fisher Scientific, Waltham, Massachusetts) and a Qubit RNA kit. RNA samples with an integrity index ≥ 7.0 and a 28S : 18S ratio ≥ 1.5 were selected for subsequent assays.

Ribosomal RNA (rRNA) was removed from each RNA (5 *μ*g) utilizing the Ribo-Zero™ rRNA Removal Kit (Epicentre Technologies, Madison, Wisconsin) and linear RNA was removed using RNase R (Epicentre Technologies). According to the manufacturer's instructions, the NEB Next Ultra RNA Library Prep Kit for Illumina (NEB) was employed to construct the libraries for sequencing. The NEBNext First-Strand Synthesis Reaction Buffer (5x) was used to fragment the RNA into pieces of approximately 300 bp in length. First-strand cDNA was synthesized using reverse transcriptase primers and random primers, and the second-strand cDNA was synthesized in second-strand synthesis reaction buffer in dUTP Mix (10x). The cDNA fragment end repair process included the addition of a single “A” base and ligation of the adapters. Once the Illumina sequencing adaptors are ligated, the USER Enzyme (NEB) was used to digest the second strand of cDNA to generate a chain-specific library. Library DNA was amplified, purified, and subjected to PCR enrichment. The library was identified by Agilent 2100 and quantified utilizing the KAPA Library Quantification Kit (KAPA Biosystems, South Africa). Finally, paired-end sequencing was processed on an Illumina NextSeq CN500 (Illumina, San Diego, CA).

### 2.7. Quality Control and Comparison

The quality of the paired-end reads of the raw sequencing data was examined using FastQC software. The raw sequencing data was preprocessed by Cutadapt software 1.18 to remove the Illumina sequencing adaptors and poly (A) tailing sequences. Reads with *N* concentration > 5% were removed by the Perl Script and those with 70% base mass > 20% were identified using the FASTX Toolkit software (v0.0.13). Both ends were repaired using the BBMap software. The high-quality fragments of the reads were mapped onto the human reference genome for comparison.

### 2.8. Isolation and Culture of Mouse Nucleus Pulposus Cells (NPCs)

NP tissues were harvested from the intervertebral discs of normal mice, cut into tiny pieces, detached with 0.25 mg/mL type II collagenase, and filtered (70 *μ*m). The isolated NPCs were cultured in DMEM-F12 (Gibco) supplemented with 1% streptomycin-penicillin (Invitrogen) and 15% fetal bovine serum (FBS) (Gibco). NPCs were immunophenotypically characterized using anti-CD24 antibody (ab31622, Abcam) and anti-KRT18 antibody (ab215839, Abcam). Cells at passage 2 were considered suitable for subsequent experiments [20].

NPCs were exposed to different concentrations (25, 50, and 100 *μ*M) of tert-butyl hydroperoxide (tBHP) (Sigma-Aldrich, St. Louis, MO) for 6 h or 12 h to establish an *in vitro* IDD cell model [21–24]. The ferroptosis-specific inhibitors ferrostatin-1 (5 *μ*M, HY-100579, MedChemExpress, Monmouth Junction, NJ), liproxstatin-1 (5 *μ*M, HY-12726, MedChemExpress), and deferoxamine (DFO) (100 *μ*M, HY-B0988, MedChemExpress) were added to the NPC culture medium. EVs (1 *μ*g/mL) were added to the NPC culture medium for coculture.

### 2.9. Isolation and Characterization of BMSC-EVs

mBMSCs (D1, CRL12424; ATCC, Manassas, VA) were cultured in DMEM-F12 basic medium (HyClone Company) replenishing 10% FBS (10099141, Gibco company) and 0.2% penicillin-streptomycin (HyClone Company) and subcultured every 3 days. Then, BMSCs were cultured in osteogenic, adipogenic, and chondrogenic differentiation OriCell MSC culture medium (Cyagen Company, Guangzhou, China), followed by differentiation ability determination utilizing alizarin red standing, oil red O staining, and alcian blue staining, respectively [25]. BMSCs were maintained in serum-free DMEM for 2 days and then centrifuged at 800 × g and 4°C for 10 min and 10000 × g for 10 min and filtered through a 40 *μ*m membrane to remove cell debris and dead cells. The supernatant was subjected to ultracentrifugation at 100000 × g and 4°C for 1 h using a SW70Ti rotor (Beckman Coulter Inc., Chaska, MN) [26]. The extracted pellets were stored at −80°C.

The morphology of the pellets was observed utilizing a transmission electron microscope (TEM) (HT7830, Hitachi, Tokyo, Japan). The particle size of the pellets was determined utilizing the NanoSight LM10 instrument (NanoSight Ltd., Minton Park, UK). Further characterization was performed with Western blot to quantify the expression patterns of the EV-specific surface markers CD63 (1 : 1000, ab134045, Abcam), calnexin (1 : 100, ab22595, Abcam), and TSG101 (1 : 1000, ab125011, Abcam).

### 2.10. Fluorescent Labeling with Cy3

BMSCs were detached with 0.25% trypsin, resuspended in DMEM with 10% FBS, and adjusted to a concentration of 1 × 10^6^ cells/well. Cy3-labeled circ_0050205 (circ_0050205-Cy3, GenePharma, Shanghai, China) was transfected into BMSCs employing the Lipofectamine 2000 reagent (11668019, Invitrogen). BMSCs expressing circ_0050205-Cy3 were seeded in 6-well plates and cocultured with NPCs in the transwell chamber for 2–4 days. The nuclei of NPCs were stained with 10 *μ*g/mL Hoechst 33342 (C1025, Beyotime, Nantong, Jiangsu, China). The NPC nuclei and circ_0050205-Cy3 expression were observed under a confocal microscope.

### 2.11. EV Uptake Assay

Upon reaching 50% confluence, NPCs were fixed with 4% paraformaldehyde for 30 min, permeabilized in 2% Triton X-100 for 15 min, and blocked with 2% BSA for 45 min. After coculture with PHK67-labeled BMSC-EVs (100 *μ*g/mL) [27] for 24 h, the NPCs were stained with 2 *μ*g/mL DAPI and the fluorescence intensity was observed under a fluorescence microscope.

### 2.12. Cell Transfection

NPCs were detached with 0.25% trypsin, seeded in 6-well plates (1 × 10^5^ cells/well), and harvested after 24 h. When reaching 75% confluence, NPCs were transduced with an empty vector, LV-circ_0050205 sh-NC, sh-circ_0050205, mimic NC, miR-665 mimic, inhibitor NC, miR-665 inhibitor, LV-circ_0050205 + mimic NC, LV-circ_0050205 + miR-665 mimic, LV-GPX4, LV-circ_0050205 + sh-NC, and LV-circ_0050205 + sh-GPX4 (50 ng/mL for each plasmid) using the Lipofectamine 2000 reagent (Invitrogen). At 12 h after transfection, the NPCs were treated with 100 *μ*M tBHP for 24 h.

### 2.13. Cell Viability Assay

NPCs were seeded at a density of 5 × 10^3^ cells/well in a 96-well plate. Upon reaching 60% confluence, the cells were exposed to different doses of tBHP and cultured for 6 or 12 h; alternatively, after 12 h of transfection, the cells were exposed to different doses of tBHP for 6, 12, 24, 48, and 72 h. Then, 10 *μ*L CCK-8 (K1018, APExBIO) was added to each well, followed by 2 h of incubation at 37°C. The absorbance (at 450 nm) was determined by a Multiskan FC reader (51119080, Thermo Fisher Scientific) to quantify cell viability.

### 2.14. Cell Proliferation Assay

NPCs were seeded at a density of 5 × 10^3^ cells/well in a 96-well plate and treated with 10 *μ*M EdU (COO81L, Beyotime) for 24 h. After fixation with paraformaldehyde, the cells were stained with Hoechst 33342. Images were captured using a fluorescence microscope (Thermo Fisher Scientific) at excitation wavelengths of 350 nm and 550 nm. The numbers of EdU-positive NPCs were counted to evaluate proliferation. The EdU staining positive rate = EdU − stained positive cells/total cells × 100%.

### 2.15. Cell Apoptosis Assay

Transfected and tBHP-exposed NPCs were incubated with a mixture (100 *μ*g/mL) of 5 *μ*L Annexin V-APC and 1 *μ*L PI for 15 min according to the kit instructions (Keygen Biotech, China). Apoptotic cells were examined using the FACScan flow system (Becton Dickinson, San Diego, CA) and analyzed employing FlowJo software.

### 2.16. ROS Production Measurement

ROS production in cells was detected by the ROS Detection Kit (CA1410, Beijing Solarbio Science & Technology Co. Ltd., Beijing, China). NPCs were incubated with 1 mL diluted dichlorodihydrofluorescein diacetate (DCF-DA) for 20 min and rinsed with serum-free medium three times to remove the excess DCF-DA that failed to enter the cells. A confocal microscope (Olympus FV10C-W3) was used to observe the cells. The fluorescence intensity was quantified at 485 nm (excitation wavelength) and 535 nm (emission wavelength) using a SpectraMax M5 reader.

### 2.17. C11-BODIPY581/591

NPCs were treated with 10 *μ*M C11-BODIPY581/591 (D3861, Thermo Fisher Scientific) for 1 h, followed by PBS washing. Cells were trypsinized and collected where lipid peroxidation was measured utilizing flow cytometry (FACSCanto™ II, BD Biosciences, Tokyo, Japan) [28].

### 2.18. Measurement of Labile Iron Levels

The iron levels were determined using a commercial kit (ab83366, Abcam). Briefly, NPCs were homogenized in a 5× volume of iron assay buffer on ice, followed by centrifugation at 13000 × g and 4°C for 10 min. The supernatant was incubated with 5 *μ*L of an iron reducer for 30 min at 37°C, followed by incubation with 100 *μ*L of an iron probe for 60 min at 37°C in the dark. The absorbance value was quantified at a wavelength of 593 nm utilizing a microplate reader (Thermo Fisher Scientific).

### 2.19. Immunofluorescence

The cell slides were blocked with 10% BSA for 1 h at 25°C and probed with primary antibodies against rabbit anti-MMP13 (ab39012, 1 : 200, Abcam, RRID: AB_776416), rabbit anti-ADAMTS 5 (ab41037, 1 : 500, Abcam, RRID: AB_2222327), rabbit anti-COL II (ab34712, 1 : 1000, Abcam, RRID: AB_731688), and rabbit anti-aggrecan (ab36861, 1 : 200, Abcam, RRID: AB_722655) for 12–16 h at 4°C. After PBS washing, the cell slides were reprobed with donkey anti-rabbit IgG labeled with Alexa Fluor 647 (Thermo Fisher Scientific) for 1 h at 25°C. The nuclei were stained with 10 *μ*g/mL DAPI (Sigma-Aldrich) for 15 min at 25°C. A fluorescence or confocal microscope (Olympus) was used to observe the slides in a blinded manner.

### 2.20. TEM

NPCs were centrifuged at 1000 × g for 15 min. The precipitates were fixed with PBS supplemented with 2.5% glutaraldehyde for 2 h and incubated with 1% osmium tetroxide for 2 h. Then, the precipitates were dehydrated with gradient alcohol and embedded with Epon 812. After ultrathin sectioning, the sections were stained with uranyl acetate and lead citrate and examined using a TEM (Tecnai G2 12, FEI Company, Netherlands).

### 2.21. Luciferase Assay

miR-665 binding sites in the circ_0050205 and GPX4 sequences were predicted using the TargetScan and DIANA TOOLS databases, respectively. Wild-type circ_0050205 (circ_0050205 WT), mutated circ_0050205 (circ_0050205 Mut), wild-type GPX4 mRNA 3′-UTR (GPX4 WT), and mutated GPX4 mRNA 3′-UTR (GPX4 Mut) were constructed and independently inserted into the pGL-3 luciferase reporter vector (RealGene, Shanghai, China). The designed pGL-3 luciferase reporter plasmids carrying the miR-665 mimic or mimic NC were cotransfected into 293T cells. After 48 h, the luciferase activity was quantified utilizing the Dual-Luciferase® Reporter Assay System (E1910, Promega, Madison, WI).

### 2.22. RNA Immunoprecipitation (RIP) Assay

The binding of miR-665 and circ_0050205 to the Ago2 protein was examined using the RIP kit (Millipore, Billerica, MA). Briefly, mononuclear cells were washed, lysed by incubation with RIPA buffer (P0013B, Beyotime) for 5 min, and centrifuged (7000 × g, 10 min) at 4°C. One part of the cell extract was used as input, and another part was used for immunoprecipitation. Briefly, 50 *μ*L magnetic beads were resuspended in 100 *μ*L RIP wash buffer and incubated with 5 *μ*g antibody (rabbit anti-human Ago2, ab186733, 1 : 50, Abcam, or rabbit anti-human IgG, ab109489, 1 : 100, Abcam) to allow binding. Following washing, the bead-antibody complex was resuspended in 900 *μ*L RIP wash buffer and incubated with 100 *μ*L cell extract overnight. Then, the bead-protein complex was collected. The immunoprecipitate and input were digested with proteinase K and subjected to RNA extraction and PCR analysis.

### 2.23. RNA Pulldown Assay

Bio-miR-665-WT and Bio-miR-665-MUT (GeneCreate, Wuhan, China) labeled with 50 nM biotin were used to transfect mononuclear cells. After 48 h, the mononuclear cells were collected and incubated with specific lysis buffer (Ambion, Austin, Texas) for 10 min. The cell lysates were incubated with M-280 streptavidin magnetic beads (S3762, Sigma-Aldrich) precoated with RNase-free BSA and yeast tRNA (TRNABAK-RO, Sigma-Aldrich). After washing with prechilled lysis buffer twice, low-salt buffer three times, and high-salt buffer once, the RNA was purified with TRIzol and analyzed by PCRto examine the enrichment of circ_0050205 and GPX4.

### 2.24. Real-Time Quantitative Polymerase Chain Reaction (RT-qPCR)

Following total RNA extraction using TRIzol reagent (16096020, Thermo Fisher Scientific), circRNA and mRNA were used to synthesize cDNA with an RT kit (RR047A, Takara, Japan). A PolyA Tail Detection Kit (B532451, Sangon) was used to generate cDNA from miRNA. RT-qPCR was performed using the SYBR® Premix Ex Taq™ II kit (DRR081, TaKaRa) and the PCR instrument (ABI 7500, ABI, Foster City, CA). U6 was used to normalize miRNA expression, and GAPDH was used to normalize circRNA and mRNA expression. The primer sequences are listed in Table S1. The results were calculated using the 2^−△△*CT*^ method.

### 2.25. Western Blot

Total protein was extracted using RIPA lysis buffer (C0481, Sigma-Aldrich) replenishing 1% complete protease and phosphatase inhibitor (Beyotime). The concentration of each protein sample was determined using the bicinchoninic acid (BCA) kit (23227, TH&Ermo). The protein was loaded on the SDS-PAGE gel and transferred to the PVDF membrane (Millipore). The membrane was blocked with 5% BSA at ambient temperature for 1 h and incubated with the primary antibodies (all from Abcam) overnight at 4°C and horseradish peroxidase-labeled goat anti-rabbit IgG (ab205718, 1 : 2000, Abcam) or goat anti-mouse IgG (ab197767, 1 : 2000, Abcam) for 1.5 h at room temperature. Afterwards, the bands were visualized using chemiluminescent solution (NCI4106, Pierce, Rockford, IL). ImageJ 1.48 software (National Institutes of Health, Bethesda, Maryland) was adopted for band analysis, and protein expression was normalized to GAPDH expression. The primary antibodies (all from Abcam) were rabbit anti-MMP13 (ab84594, 1 : 1000), rabbit anti-ADAMTS 5 (ab41037, 1 : 500), rabbit anti-COL II (ab188570, 1 : 1000), rabbit anti-aggrecan (ab3778, 1 : 1000), and rabbit anti-GAPDH (ab9485, 1 : 2500).

### 2.26. Statistical Analysis

Measurement data represented the results of at least three independent experiments and were described as mean ± standard deviation. Unpaired *t*-test was adopted for comparison between the two groups; one-way or two-way ANOVA was employed for data comparison among multiple groups and multiple time points, with the Tukey's test used for the post hoc test. A *P* value < 0.05 indicated significant difference. All statistical analyses were started utilizing SPSS 21.0 statistical software (IBM, Armonk, NY).

## 3. Results

### 3.1. Ferroptosis Was Associated with NPC Viability Reduction and Extracellular Matrix (ECM) Degradation Caused by Oxidative Stress (OS)

Previous evidence has shown that superoxide-triggered cell death is related to ferroptosis and OS and ECM degradation are involved in the pathogenesis of IDD [29–31]. Here, we were interested in whether ferroptosis participated in the NPC death and ECM degradation observed in IDD. For this purpose, we used tBHP to treat NPCs and performed CCK-8 and EdU assays to examine NPC proliferation. As shown in Supplementary Figures 1(a) and 1(b), NPC proliferation decreased in a concentration-dependent manner when the NPCs were exposed to 25, 50, and 100 *μ*M of tBHP for 6 h or 12 h. A concentration-dependent increase was also noted in NPC apoptosis after exposure to different concentrations of tBHP (Supplementary Figure 1c). In addition, increased expression of two catabolic markers MMP13 and ADAMTS 5 and decreased expression of two anabolic markers COL II and aggrecan were observed in NPCs exposed to tBHP stimulation (Supplementary Figure 1d). These results suggested that superoxide could lead to decreased NPC viability, NPC death, and ECM degradation.

Ferroptosis is characterized by ROS production and free Fe^2+^augmentation [32]. As shown in Supplementary Figure 1e-g, tBHP stimulation resulted in increased ROS production, lipid peroxidation, and total free iron in NPCs in a concentration-dependent manner. Subsequently, we treated NPCs with the ferroptosis-specific inhibitors Fer-1, Lip-1, and DFO. As shown by CCK-8 and EdU assays, Fer-1, Lip-1, or DFO treatment rescued the tBHP-induced decrease in NPC viability (Supplementary Figure 1h, 1i). Flow cytometric analysis (Supplementary Figure 1j) and Western blotting (Supplementary Figure 1k) demonstrated that treatment of ferroptosis-specific inhibitors weakened the effects of tBHP stimulation on NPC apoptosis and expression of catabolic and anabolic markers. Similarly, tBHP-induced increase in the ROS production, lipid peroxidation, and total free iron was abrogated in NPCs upon treatment of ferroptosis-specific inhibitors (Supplementary Figure 1l-n). TEM observation showed that tBHP stimulation led to mitochondrial damage, such as mitochondrial shrinkage, mitochondrial crista disintegration, increased mitochondrial membrane density, and ferroptotic characteristics (Supplementary Figure 1o). These data showed that ferroptosis was involved in OS and ECM degradation, which was associated with the pathogenesis of IDD.

### 3.2. BMSC-EVs Promoted the NPC Survival and Suppressed ECM Degradation in NPCs

**Figure 1:**
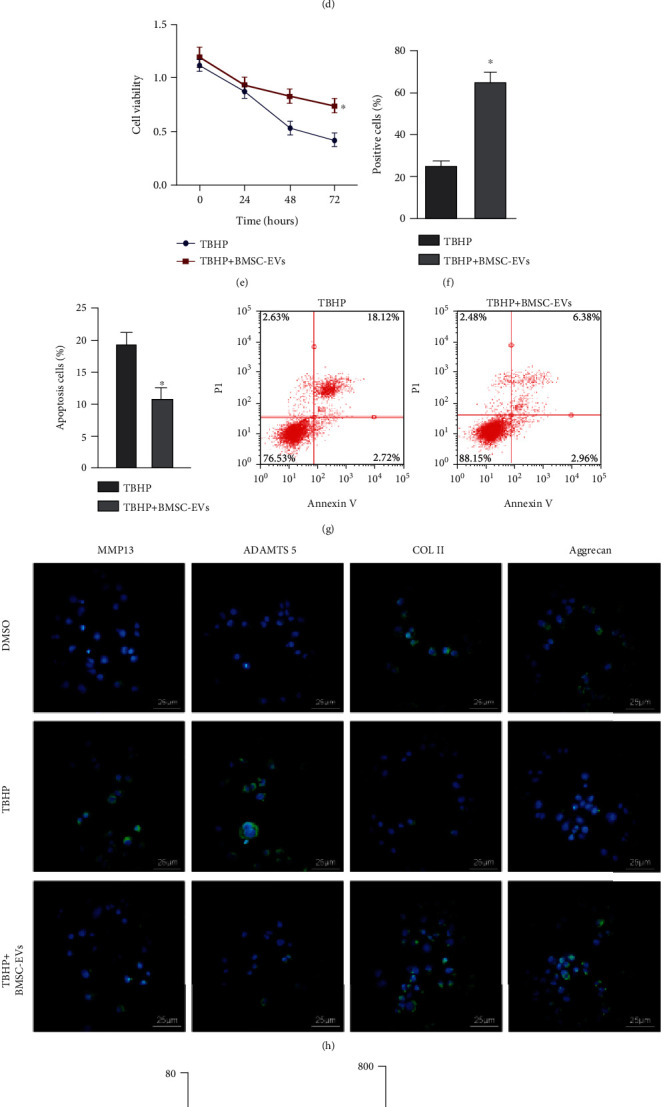
BMSC-EVs enhance the survival of NPCs and inhibit ECM degradation in NPCs. (a) Characterization of BMSC-EVs by TEM observation (100 nm). (b) NanoSight analysis of the size of BMSC-EVs in diameter. (c) Western blot of EV-specific surface markers CD63, calnexin, and TSG101 in BMSC-EVs, ^∗^*P* < 0.05 vs. the EV group. (d) NPC uptake of PKH67-labeled BMSC-EVs by NPCs (25 *μ*m). (e) CCK-8 assay for viability of 50 *μ*M tBHP-exposed NPCs for 12 h following coculture with BMSC-EVs. (f) EdU staining for proliferation of 50 *μ*M tBHP-exposed NPCs for 12 h following coculture with BMSC-EVs. (g) Flow cytometric analysis of apoptosis of 50 *μ*M tBHP-exposed NPCs for 12 h following coculture with BMSC-EVs. (h) Immunofluorescence detection of MMP13, ADAMTS 5, COL II, and aggrecan proteins in 50 *μ*M tBHP-exposed NPCs for 12 h following coculture with BMSC-EVs. (i) The ROS production was examined using DCF-DA kit in 50 *μ*M tBHP-exposed NPCs for 12 h following coculture with BMSC-EVs (25 *μ*m). (j) Lipid peroxidation determined by C11-BODIPY581/591 in 50 *μ*M tBHP-exposed NPCs for 12 h following coculture with BMSC-EVs. (k) The total free iron in 50 *μ*M tBHP-exposed NPCs for 12 h following coculture with BMSC-EVs. ^∗^*P* < 0.05 vs. the tBHP group. Cell experiments were conducted three times independently.

BMSCs have been reported to prevent IDD progression via the transfer of EVs [33, 34]. In addition, BMSCs have been found to inhibit ferroptosis [35]. Therefore, we hypothesized that BMSC-EVs could inhibit IDD progression by augmenting survival and reducing ECM degradation in NPCs. Observation results of the morphology of BMSCs under a light microscope showed that BMSCs have a long-shuttle shape or are spindle shaped (Supplementary Figure 2A). After differentiation induction under different conditions, red calcium nodules, red lipids contained in cells, and blue collagen staining appeared (Supplementary Figure 2B), indicating that BMSCs had theosteogenic, adipogenic, and chondrogenic differentiation potentials. In addition, Figure 1(a) reflects the characterization of BMSC-EVs by TEM observation and Figure 1(b) shows the size of the BMSC-EVs, with diameters ranging from 30 to 150 nm. As shown in Figure 1(c), CD63 and TSG101 were expressed in the BMSC-EVs while calnexin was not expressed. The abovementioned results indicated the successful EV extraction. Microscopic observation demonstrated NPC uptake of PKH67-labeled BMSC-EVs (Figure 1(d)).

Next, BMSC-EVs were cocultured with the 50 *μ*M tBHP-exposed NPCs for 12 h. The results of CCK-8 assay (Figure 1(e)), EdU staining (Figure 1(f)), and flow cytometric analysis (Figure 1(g)) showed that BMSC-EVs enhanced NPC viability and proliferation but reduced apoptosis under tBHP stimulation. Immunofluorescence detection demonstrated decreased expression of MMP13 and ADAMTS 5 and elevated expression of COL II and aggrecan in tBHP-exposed NPCs following the addition of BMSC-EVs (Figure 1(h)). As expected, a significant decrease was noted in ROS production, lipid peroxidation, and total free iron in tBHP-exposed NPCs following the addition of BMSC-EVs (Figures 1(i)–1(k)). These data proved that BMSC-EVs favored NPC survival and repressed ECM degradation in NPCs.

### 3.3. BMSC-EVs Carried circ_0050205 to Enhance NPC Survival and Inhibit ECM Degradation in NPCs

**Figure 2:**
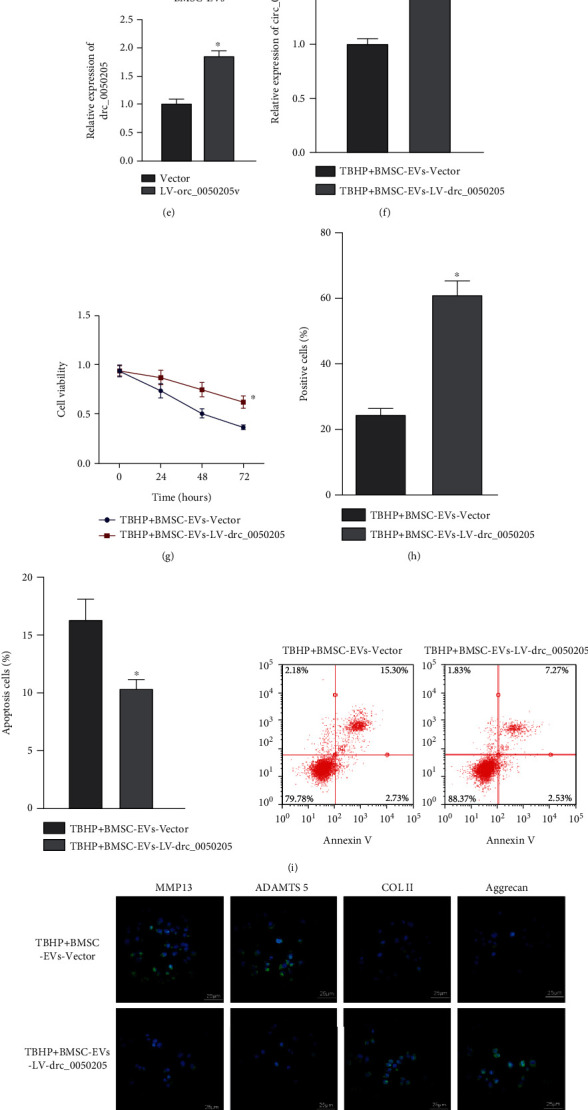
BMSC-EVs stimulated the survival of NPCs and inhibit ECM degradation in NPCs by transferring circ_0050205. (a) RT-qPCR analysis of circ_0050205 expression in the NP tissues of IDD mice (*n* = 8) and sham-operated mice (*n* = 8), ^∗^*P* < 0.05 vs. sham-operated mice. (b) RT-qPCR analysis of circ_0050205 expression in tBHP-exposed NPCs, ^∗^*P* < 0.05 vs. tBHP-0 *μ*M. (c) Transfer of circ_0050205-Cy3 from BMSCs into NPCs via EVs (25 *μ*m). (d) RT-qPCR analysis of circ_0050205 expression in tBHP-exposed NPCs cocultured with BMSC-EVs, ^∗^*P* < 0.05 vs. the tBHP group. (e) RT-qPCR analysis of circ_0050205 expression in EVs derived from circ_0050205-overexpressing BMSCs, ^∗^*P* < 0.05 vs. vector. (f) RT-qPCR analysis of circ_0050205 expression in tBHP-exposed NPCs cocultured with EVs derived from circ_0050205-overexpressing BMSCs, ^∗^*P* < 0.05 vs. tBHP + BMSC-EVs + vector. (g) CCK-8 assay for the viability of tBHP-exposed NPCs cocultured with EVs circ_0050205-overexpressing BMSCs, ^∗^*P* < 0.05 vs. tBHP + BMSC-EVs + vector. (h) EdU staining for proliferation of tBHP-exposed NPCs cocultured with EVs from circ_0050205-overexpressing BMSCs, ^∗^*P* < 0.05 vs. tBHP + BMSC-EVs + vector. (i) Flow cytometric analysis of apoptosis of tBHP-exposed NPCs cocultured with EVs from circ_0050205-overexpressing BMSCs, ^∗^*P* < 0.05 vs. tBHP + BMSC-EVs + vector. (j) Immunofluorescence detection of MMP13, ADAMTS 5, COL II, and aggrecan proteins in tBHP-exposed NPCs cocultured with EVs from circ_0050205-overexpressing BMSCs (25 *μ*m). (k) The ROS production was examined using the DCF-DA kit in tBHP-exposed NPCs cocultured with EVs from circ_0050205-overexpressing BMSCs, ^∗^*P* < 0.05 vs. tBHP + BMSC-EVs + Vector. (l) Lipid peroxidation determined by C11-BODIPY581/591 in tBHP-exposed NPCs cocultured with EVs from circ_0050205-overexpressing BMSCs. (m) The total free iron in tBHP-exposed NPCs cocultured with EVs from circ_0050205-overexpressing BMSCs, ^∗^*P* < 0.05 vs. tBHP + BMSC-EVs + vector. ^∗^*P* < 0.05. Cell experiments were conducted three times independently.

To study the mechanism underlying the effects of BMSC-EVs on NPC survival and ECM degradation, we performed RNA sequencing, with screened 337 upregulated circRNAs and 283 downregulated circRNAs in the NP tissues collected from mice with IDD (Supplementary Figure 3a-b). Among these circRNAs, circ_0050205 exhibited the largest fold change in expression. The results of RT-qPCR showed that circ_0050205 expression was downregulated in the NP tissues of IDD mice compared to that of sham-operated mice (Figure 2(a)). Similarly, we observed a declined expression of circ_0050205 in NPCs after tBHP stimulation (Figure 2(b)).

Next, BMSC-EVs expressing Cy3-circ_0050205 were allowed to coculture with NPCs. As depicted in Figure 2(c), Cy3-circ_0050205 was observed in the NPCs, suggesting that circ_0050205 was transferred from BMSCs into NPCs via EVs. RT-qPCR revealed an elevated expression of circ_0050205 in tBHP-exposed NPCs after coculture with BMSC-EVs (Figure 2(d)). These data suggested that circ_0050205 can be transferred from BMSCs into NPCs via EVs.

Further investigation was performed to investigate whether circ_0050205 mediated the effects of BMSC-EVs on NPC survival and ECM degradation in NPCs. We generated circ_0050205-overexpressing BMSCs by the delivery of the circ_0050205 expression vector (Figure 2(e)). Then, we isolated EVs from circ_0050205-overexpressing BMSCs and cocultured them with NPCs in the presence of tBHP. RT-qPCR results demonstrated elevated expression of circ_0050205 in tBHP-exposed NPCs cocultured with EVs from circ_0050205-overexpressing BMSCs (Figure 2(f)). Results of CCK-8 assay (Figure 2(g)), EdU staining (Figure 2(h)), and flow cytometric analysis (Figure 2(i)) showed that EVs derived from circ_0050205-overexpressing BMSCs could promote the viability and proliferation and inhibit the apoptosis of tBHP-exposed NPCs. Immunofluorescence detection demonstrated decreased expression of MMP13 and ADAMTS 5 yet elevated expression of COL II and aggrecan in tBHP-exposed NPCs cocultured with EVs derived from circ_0050205-overexpressing BMSCs (Figure 2(j)). We also found a significant decrease in ROS production, lipid peroxidation production, and total free iron in tBHP-exposed NPCs cocultured with EVs derived from circ_0050205-overexpressing BMSCs (Figures 2(k)–2(m)). These results collectively indicated that BMSC-EVs facilitated NPC survival and inhibited ECM degradation in NPCs by transferring circ_0050205.

### 3.4. circ_0050205 Sponged miR-665 to Upregulate GPX4 Expression

**Figure 3:**
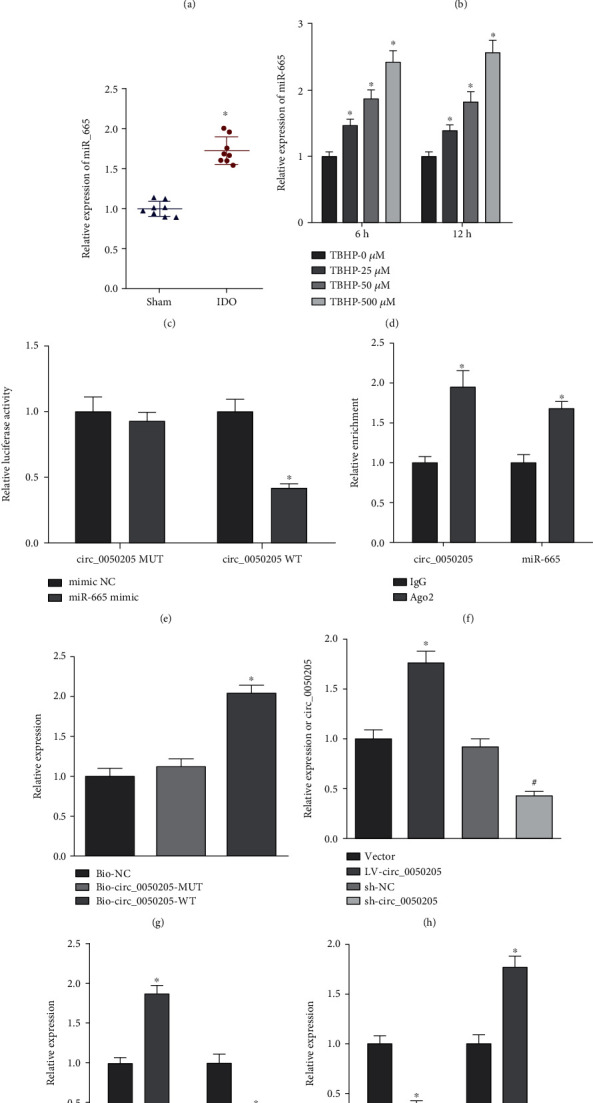
circ_0050205 functions as a ceRNA to sponge miR-665. (a) Venn diagram showing common downstream miRNAs of circ_0050205 in the Circbank and CircInteractome databases. (b) The binding sites between circ_0050205 and miR-665. (c) miR-665 expression in mouse NP tissues of IDD mice (*n* = 8) and sham-operated mice (*n* = 8) determined by RT-qPCR, ^∗^*P* < 0.05 vs. sham-operated mice. (d) miR-665 expression in tBHP-exposed NPCs determined by RT-qPCR, ^∗^*P* < 0.05 vs. tBHP 0 *μ*M. (e) circ_0050205 binding with miR-665 was examined by luciferase activity assay, ^∗^*P* < 0.05*vs.* mimic NC. (f) Enrichment of circ_0050205 and miR-665 in the immunoprecipitates using anti-Ago2 or IgG, ^∗^*P* < 0.05 vs. IgG. (g) RNA pulldown demonstrated that more miR-665 was pulled down using Bio-circ_0050205-WT and Bio-circ_0050205-Mut, ^∗^*P* < 0.05*vs.* Bio-miR-665-Mut. (h) circ_0050205 overexpression or knockdown efficiency in NPCs determined by RT-qPCR, ^∗^*P* < 0.05 vs. vector, ^#^*P* < 0.05 vs. sh-NC. (i) RT-qPCR analysis of circ_0050205 and miR-665 expression in tBHP-exposed NPCs treated with LV-circ_0050205 or sh-circ_0050205. ^∗^*P* < 0.05*vs.* tBHP + vector or tBHP + sh-NC. Cell experiments were conducted three times independently.

Subsequently, we performed a computer-based prediction of interactions between circ_0050205 and its downstream miRNAs in the Circbank and CircInteractome databases. These two databases yielded 4 common downstream miRNAs (miR-665, miR-1248, miR-1272, and miR-1265) of circ_0050205 (Figures 3(a) and 3(b)). Considering the upregulated miR-665 expression observed in the context of IDD from previous evidence [36], miR-665 was selected for further investigation. We also observed increased miR-665 expression in the NP tissues of IDD mice compared to sham-operated mice (Figure 3(c)). Similarly, upregulated miR-665 expression was observed in tBHP-exposed NPCs (Figure 3(d)). Luciferase assay demonstrated reduced luciferase activity of circ_0050205-WT after miR-665 mimic transfection, while the luciferase activity of circ_0050205-Mut did not change (Figure 3(e)). In addition, RIP assay demonstrated significant enrichments of circ_0050205 and miR-665 in the immunoprecipitates using anti-Ago2 compared with IgG (Figure 3(f)). Meanwhile, RNA pulldown assay demonstrated that more miR-665 was pulled down utilizing Bio-circ_0050205-WT than Bio-circ_0050205-Mut (Figure 3(g)). The interaction between circ_0050205 and miR-665 was further confirmed by RT-qPCR. We delivered the circ_0050205 expression vector or sh-circ_0050205 into NPCs and constructed circ_0050205-overexpressing or circ_0050205-knockdown NPCs (Figure 3(h)). Decreased expression of miR-665 was observed in tBHP-treated NPCs after circ_0050205 overexpression, while the opposite trend was observed aftercirc_0050205 knockdown (Figure 3(i)).

**Figure 4:**
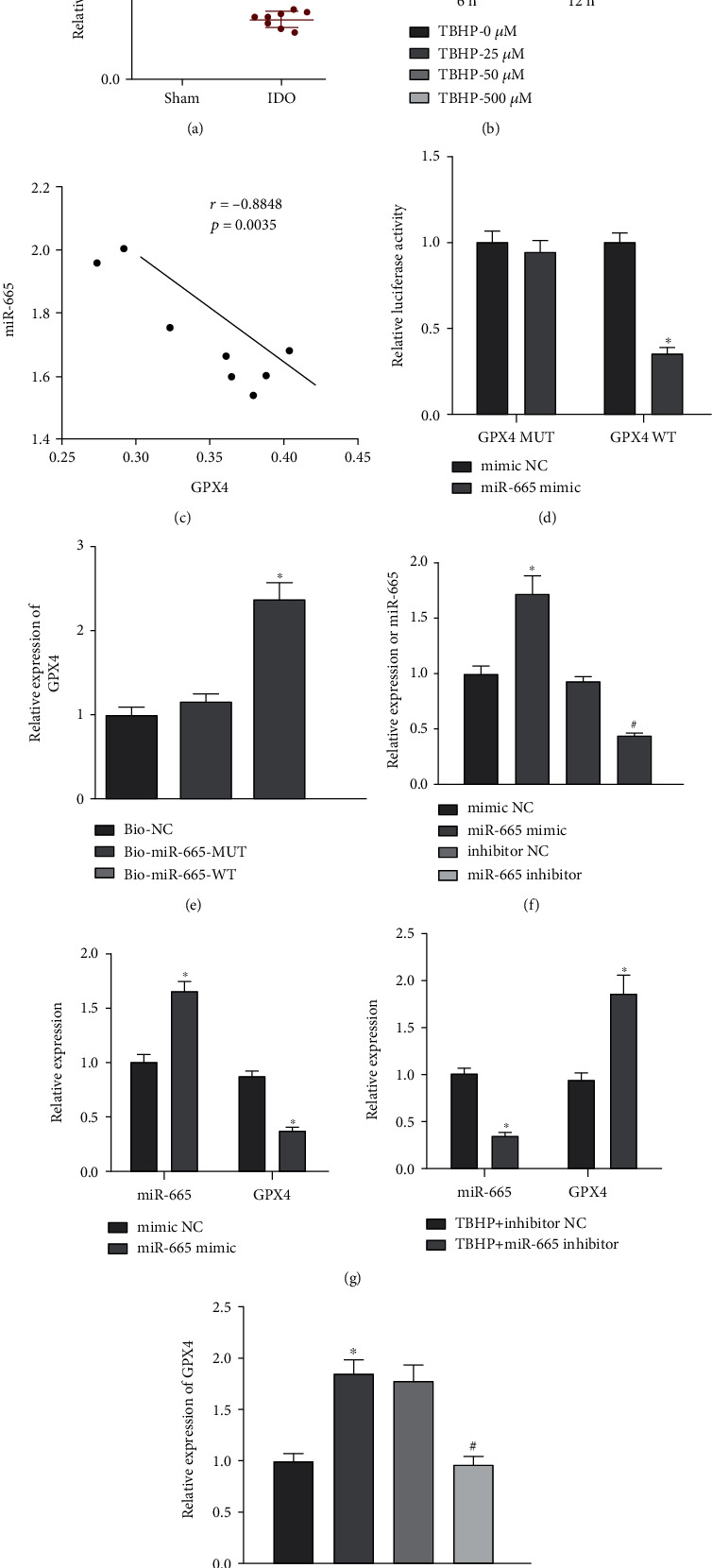
circ_0050205upregulates GPX4 expression by acting as a asmiR-665 sponge. (a) GPX4expression in mouse NP tissues of IDD mice (*n* = 8) and sham-operated mice (*n* = 8) determined by RT-qPCR, ^∗^*P* < 0.05 vs. sham-operated mice. (b) GPX4 expression in tBHP-exposed NPCs determined by RT-qPCR, ^∗^*P* < 0.05 vs. tBHP 0 *μ*M. (c) Pearson correlation analysis of miR-665 expression and GPX4 expression in the NP tissues of IDD mice (*n* = 8). (d) miR-665 binding to GPX4 examined by luciferase activity assay, ^∗^*P* < 0.05*vs.* mimic NC. (e) RNA pulldown demonstrated that Bio-miR-665-WT bound more GPX4 than Bio-miR-665-Mut, ^∗^*P* < 0.05 vs. Bio-miR-665-MUT. (f) miR-665 expression in NPCs following miR-665 mimic and inhibitor transfection determined by RT-qPCR. ^∗^*P* < 0.05 vs. mimic NC, ^#^*P* < 0.05*vs.* inhibitor NC. (g) RT-qPCR analysis of GPX4 expression in tBHP-exposed NPCs after miR-665 overexpression or inhibition, ^∗^*P* < 0.05 vs. tBHP + mimic NC or tBHP + inhibitor NC. (h) RT-qPCR analysis of GPX4 expression in tBHP-exposed NPCs treated with LV-circ_0050205 or combined with miR-665 mimic. ^∗^*P* < 0.05 vs. tBHP + vector, ^#^*P* < 0.05 vs. tBHP + LV-circ_0050205 + mimic NC. Cell experiments were conducted three times independently.

Next, computer-based miRNA-mRNA prediction was performed with the RNA22 database and the miR-665 binding sites in the GPX4 3'UTR were found (Supplementary Figure 3c). The KEGG pathway analysis found that GPX4 was part of the ferroptosis regulatory network (Supplementary Figure 3d). As shown in Figures 4(a) and 4(b), GPX4 was poorly expressed in the NP tissues of IDD mice and tBHP-exposed NPCs. Pearson correlation analysis demonstrated a negative correlation between miR-665 expression and GPX4 expression in the NP tissues of IDD mice (Figure 4(c)). Results of luciferase assay found that the luciferase activity of GPX4-WT was reduced after miR-665 mimic transfection, while there was no alteration in the luciferase activity of GPX4-Mut (Figure 4(d)). RNA pulldown assay data demonstrated that Bio-miR-665-WT bound more GPX4 than Bio-miR-665-Mut (Figure 4(e)). To further study whether GPX4 was a target gene of miR-665, we treated NPCs with the miR-665 mimic, inhibitor, and their corresponding NCs, followed by tBHP stimulation (Figure 4(f)). Elevated expression of miR-665 and declined expression of GPX4 were noted in tBHP-exposed NPCs upon miR-665 mimic transfection while opposing tendency was found in tBHP-exposed NPCs upon miR-665 inhibitor transfection (Figure 4(g)). These results suggested that GPX4 was a target gene of miR-665.

Finally, we aimed to study the regulatory network that includes circ_0050205, miR-665, and GPX4. We observed increased GPX4 expression in tBHP-exposed NPCs upon circ_0050205 overexpression. When the circ_0050205 expression vector and miR-665 mimic were concomitantly transfected into NPCs in the presence of tBHP, we observed a decrease in GPX4 expression compared with LV-circ_0050205 transfection alone (Figure 4(h)). These results collectively revealed that circ_0050205 functioned as a ceRNA to sponge miR-665 and then upregulated GPX4 expression.

### 3.5. GPX4 Overexpression Enhanced NPC Survival and Retarded ECM Degradation in NPCs

**Figure 5:**
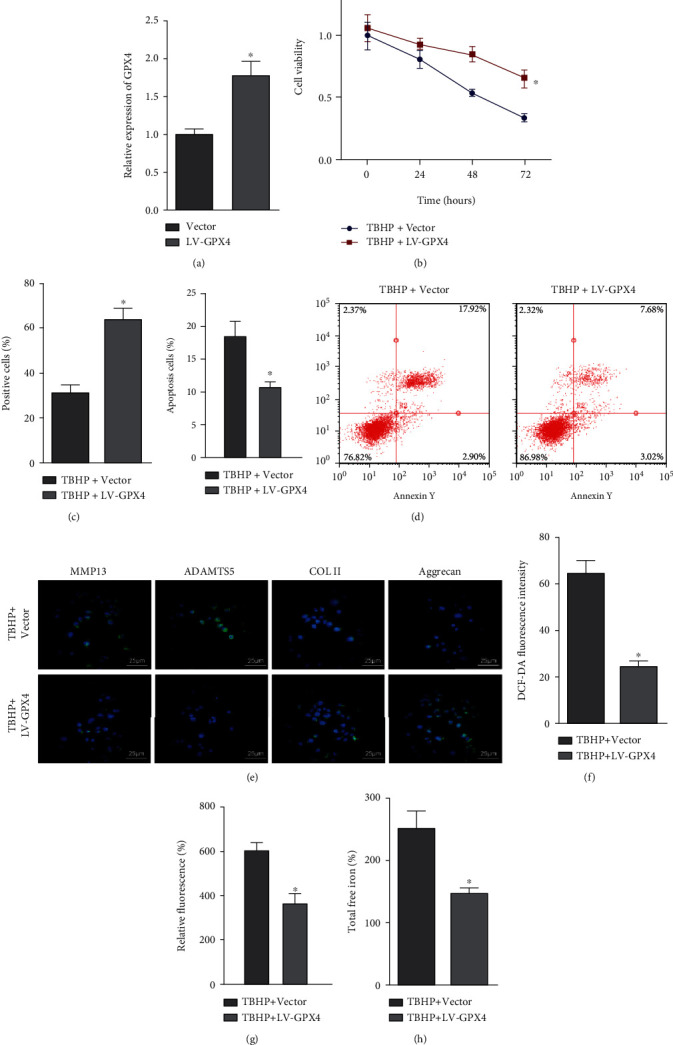
GPX4 contributes to the survival of NPCs and reduces ECM degradation in NPCs. (a) GPX4 overexpression efficiency in NPCs confirmed by RT-qPCR. (b) CCK-8 assay of viability of tBHP-exposed NPCs treated with LV-GPX4. (c) EdU staining of proliferation of tBHP-exposed NPCs treated with LV-GPX4. (d) Flow cytometric analysis of apoptosis of tBHP-exposed NPCs treated with LV-GPX4. (e) Immunofluorescence detection of MMP13, ADAMTS 5, COL II, and aggrecan proteins in tBHP-exposed NPCs treated with LV-GPX4 (25 *μ*m). (f) The ROS production was examined using the DCF-DA kit in tBHP-exposed NPCs treated with LV-GPX4. (g) Lipid peroxidation determined by C11-BODIPY581/591 in tBHP-exposed NPCs treated with LV-GPX4. (h) The total free iron in tBHP-exposed NPCs treated with LV-GPX4. ^∗^*P* < 0.05 vs. tBHP + vector. Cell experiments were conducted three times independently.

Since GPX4 expression was downregulated when ferroptosis occurred [7], we attempted to explore the effects of GPX4 on NPC survival and ECM degradation in NPCs in the following analyses. For this purpose, we constructed GPX4-overexpressed NPCs (Figure 5(a)). The results of CCK-8 assay (Figure 5(b)), EdU staining (Figure 5(c)), and flow cytometric analysis (Figure 5(d)) showed that GPX4 overexpression could promote the viability and proliferation and inhibit the apoptosis of NPCs against tBHP stimulation. The immunofluorescence results suggested that MMP13 and ADAMTS 5 expression was declined but COL II and aggrecan expression was increased in tBHP-exposed NPCs upon GPX4 overexpression (Figure 5(e)). Additionally, a significant decrease in ROS production, lipid peroxidation production, and total free iron was observed in tBHP-exposed NPCs upon GPX4 overexpression (Figures 5(f)–5(h)). Overall, GPX4 overexpression promoted NPC survival and inhibited ECM degradation in NPCs.

### 3.6. circ_0050205 in BMSC-EVs Contributed to NPC Survival and Suppressed ECM Degradation in NPCs

**Figure 6:**
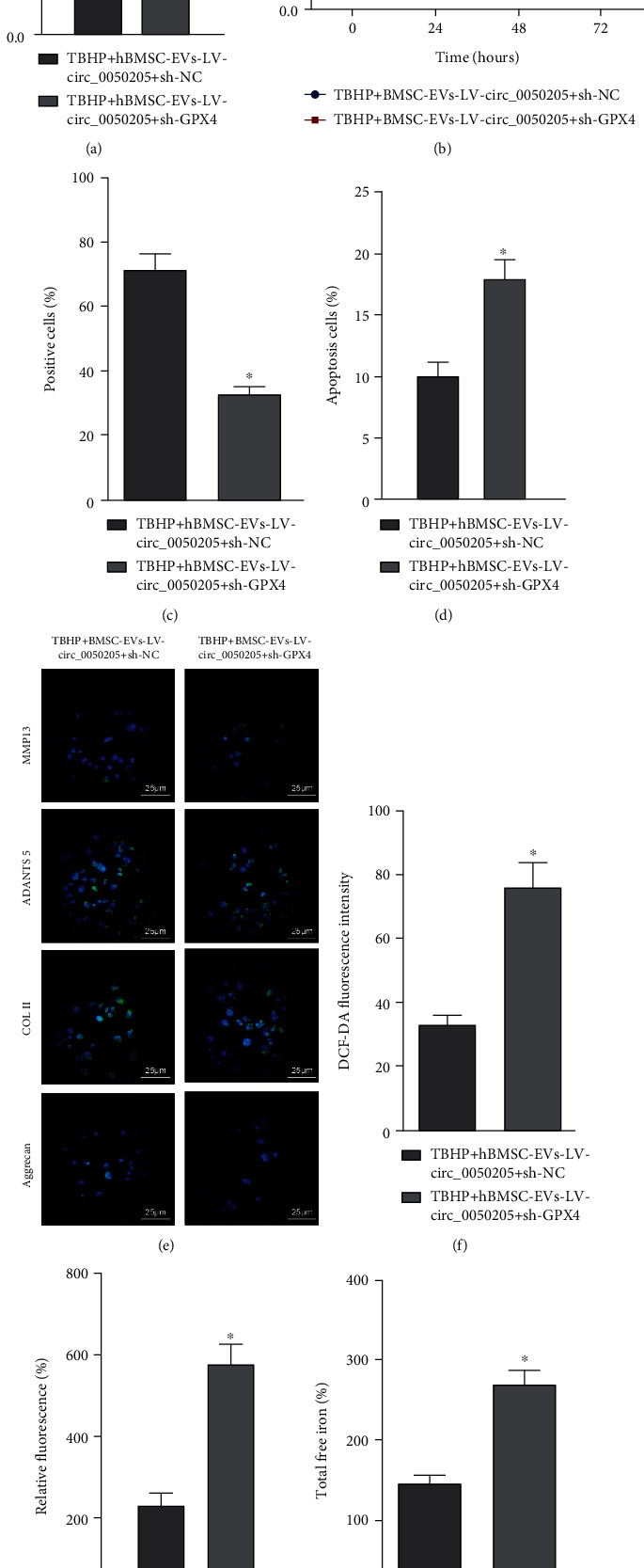
circ_0050205 in BMSC-EVs contributes to the survival of NPCs and inhibits ECM degradation in NPCs via the miR-665/GPX4 axis. (a) GPX4 expression in tBHP-exposed NPCs cocultured with BMSC-EVs-LV-circ_0050205 + sh-GPX4. (b) CCK-8 assay for viability of tBHP-exposed NPCs cocultured with BMSC-EVs-LV-circ_0050205 + sh-GPX4. (c) EdU staining for proliferation of tBHP-exposed NPCs cocultured with BMSC-EVs-LV-circ_0050205 + sh-GPX4. (d) Flow cytometric analysis of apoptosis of tBHP-exposed NPCs cocultured with BMSC-EVs-LV-circ_0050205 + sh-GPX4. (e) Immunofluorescence detection of MMP13, ADAMTS 5, COL II, and aggrecan proteins in tBHP-exposed NPCs cocultured with BMSC-EVs-LV-circ_0050205 + sh-GPX4 (25 *μ*m). (f) The ROS production was examined using DCF-DA kit in tBHP-exposed NPCs cocultured with BMSC-EVs-LV-circ_0050205 + sh-GPX4. (g) Lipid peroxidation determined by C11-BODIPY581/591 in tBHP-exposed NPCs cocultured with BMSC-EVs-LV-circ_0050205 + sh-GPX4. (h) The total free iron in tBHP-exposed NPCs cocultured with BMSC-EVs-LV-circ_0050205 + sh-GPX4. ^∗^*P* < 0.05 vs. tBHP + BMSC-EVs + LV-circ_0050205 + sh-NC. Cell experiments were conducted three times independently.

To confirm that the BMSC-EVs carrying circ_0050205 mediated the regulatory effects of miR-665 on GPX4 expression in NPCs, BMSC-EVs were added to NPCs and GPX4 expression was inhibited. In the tBHP-induced NPCs, compared with NPCs cocultured with EVs derived from BMSCs transduced with LV-circ_0050205 alone, NPCs cocultured with EVs derived from BMSCs transduced with sh-GPX4 exhibited downregulated GPX4 expression (Figure 6(a)). The results of CCK-8 assay and EdU staining revealed that GPX4 knockdown resulted in decreased viability and proliferation in NPCs cultured with EVs derived from circ_0050205-overexpressing BMSCs (Figures 6(b) and 6(c)). Flow cytometric analysis (Figure 6(d)) also demonstrated that GPX4 knockdown induced the apoptosis in NPCs cocultured with EVs derived from circ_0050205-overexpressing BMSCs.

As evidenced by immunofluorescence detection, GPX4 knockdown increased MMP13 and ADAMTS 5 expression but decreased COL II and aggrecan expression in tBHP-induced NPCs conditioned with EVs derived from circ_0050205-overexpressing BMSCs (Figure 6(e)). Remarkable increases in ROS production, lipid peroxidation production, and total free iron were observed in tBHP-induced NPCs conditioned with EVs derived from circ_0050205-overexpressing BMSCs once sh-GPX4 was transfected (Figures 6(f)–6(h)). The abovementioned data revealed that circ_0050205 delivered by BMSC-EVs augmented NPC survival and inhibited ECM degradation in NPCs via the miR-665/GPX4 axis.

### 3.7. circ_0050205 In BMSC-EVs Attenuated IDD Progression via the miR-665/GPX4 Axis

**Figure 7:**
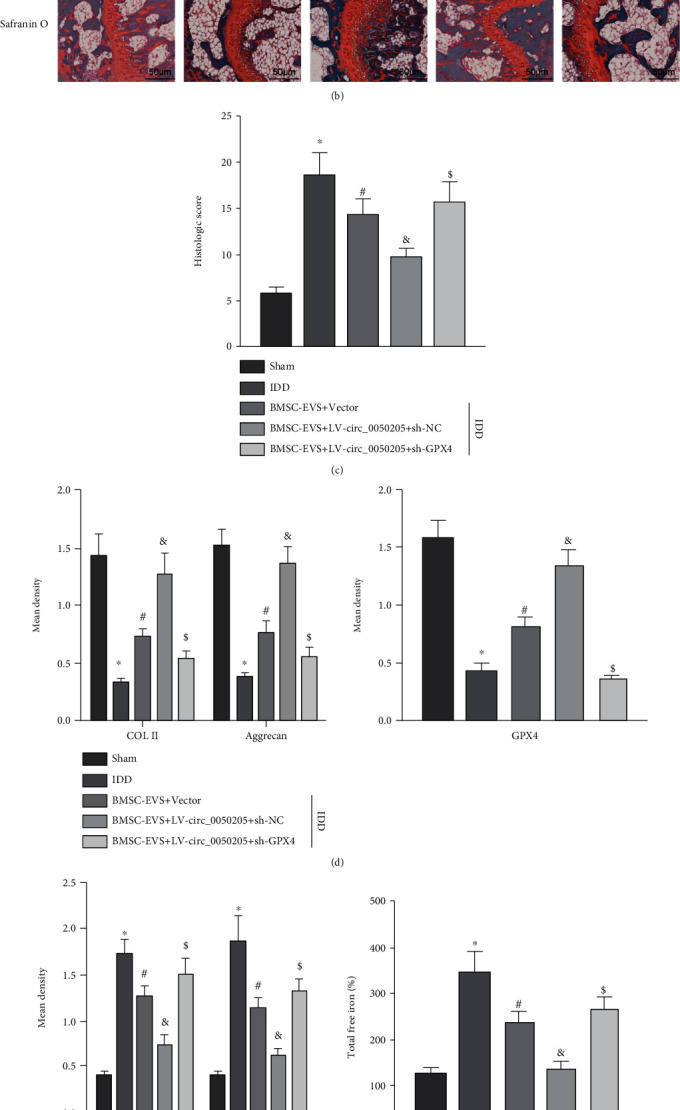
circ_0050205 in BMSC-EVs attenuates IDD progression by regulating the miR-665/GPX4 axis. A total of 40 C57BL/6J male mice were randomly assigned into the sham group, IDD group, BMSC-EV + vector group, BMSC-EV + LV-circ_0050205 + sh-NC group, and BMSC-EV + LV-circ_0050205 + sh-GPX4 group, with 8 mice per group. (a) X-ray examination of degenerative characteristics of IDD mice and DHI (%). (b) Histological examinations of mouse NP tissues by HE staining and oil red O staining (50 *μ*m). (c) Histological scores. (d) Immunohistochemical staining of COL II, aggrecan, and GPX4 proteins in mouse NP tissues. (e) Immunohistochemical staining of MMP13 and ADAMTS 5 proteins in mouse NP tissues. (f) The total free iron in mouse NP tissues. (g) RT-qPCR analysis of circ_0050205, miR-665, and GPX4 expression in mouse NP tissues. ^∗^*P* < 0.05 vs. sham, ^#^*P* < 0.05 vs. IDD, ^&^*P* < 0.05 vs. BMSC-EVs + vector, ^$^*P* < 0.05 vs. BMSC-EVs + LV-circ_0050205 + sh-NC.

To confirm the therapeutic role of BMSC-EVs in IDD, IDD was surgically established in C57BL/6J mice. Then, IDD mice were treated with EVs derived from circ_0050205-overexpressing BMSCs and/or adenovirus containing sh-GPX4. X-ray examination (Figure 7(a)) 8 weeks after surgery showed that compared with sham-operated mice, IDD mice presented degenerative characteristics. When IDD mice were injected with BMSC-EVs or EVs derived from circ_0050205-overexpressing BMSCs, the degeneration was attenuated. When IDD mice were injected with EVs derived from circ_0050205-overexpressing BMSCs and adenovirus containing sh-GPX4, GPX4 knockdown negated the inhibitory effects of circ_0050205 overexpression on IDD.

Similarly, histological examinations by HE staining and oil red O staining confirmed the X-ray examination (Figures 7(b) and 7(c)). As shown in Figures 7(d) and 7(e), compared with sham-operated mice, IDD mice exhibited reduced immunohistochemical staining of COL II, aggrecan, and GPX4 concomitant with increased immunohistochemical staining of MMP13 and ADAMTS 5. After injection of BMSC-derived EVs or EVs derived from circ_0050205-overexpressing BMSCs, IDD mice showed increased immunohistochemical staining of COL II, aggrecan, and GPX4 with reduced immunohistochemical staining of MMP13 and ADAMTS 5. Similarly, injection of adenovirus containing sh-GPX4 negated the effects of EVs derived from circ_0050205-overexpressing BMSCs on the expression of these markers.

It was found that IDD mice exhibited increased total free iron compared with sham-operated mice. Injection of BMSC-derived EVs or EVs derived from circ_0050205-overexpressing BMSCs reduced total free iron in IDD mice, while injection of adenovirus containing sh-GPX4 negated the inhibitory effects of EVs derived from circ_0050205-overexpressing BMSCs on total free iron (Figure 7(f)).

RT-qPCR (Figure 7(g)) revealed reduced expression of circ_0050205 and GPX4 concomitant with an elevated expression of miR-665 in IDD mice compared with sham-operated mice. Injection of BMSC-derived EVs or EVs derived from circ_0050205-overexpressing BMSCs increased circ_0050205 and GPX4 expression but declined miR-665 in IDD mice. GPX4 was knocked down when IDD mice were injected with EVs derived from circ_0050205-overexpressing BMSCs and adenovirus containing sh-GPX4. Altogether, BMSC-EVs carrying circ_0050205 regulate NPC survival via the miR-665/GPX4 axis, thus alleviating IDD in mice.

## 4. Discussion

MSC-based cell strategies have been developed to promote NPC regeneration in IDD. BMSCs secrete EVs that can shuttle their cargos, such as circRNAs, between cells. Many circRNAs act importantly in the pathogenesis of IDD through acting as sponges for miRNAs [37]. As the obtained findings demonstrated, circ_0050205, a circRNA of interest, could be transferred from BMSCs into NPCs via EVs and then increase NPC survival and inhibit ECM degradation by binding to miR-665 and upregulating GPX4 expression.

EVs were isolated from BMSCs and added to NPC culture medium in the presence of tBHP. The promoting effects of BMSC-EVs on NPC survival were subsequently observed. Accumulating evidence shows that OS contributes to the pathogenesis of IDD [38] and ferroptosis is a form of cell death that is associated with OS and inflammation [39]. NPC ferroptosis is attracting more attention because of its role in contributing to the pathogenesis of disc degeneration. Consistent with our results, Sun et al. demonstrated that BMSCs exert protective effects on liver grafts donated after circulatory death by inhibiting hepatocyte ferroptosis [35]. In addition, Yang and his team provided evidence that EVs secreted by vascular endothelial cells inhibit glucocorticoid-induced osteoporosis by reducing ferritinophagy and inhibiting the ferroptosis of osteoblasts [40]. EVs derived from adipose-derived stem cells inhibit ferroptosis in mice with intracerebral hemorrhage [41]. In addition, we also found that BMSC-EVs could prevent ECM degradation in NPCs, as evidenced by decreased expression of two catabolic markers MMP13 and ADAMTS 5 concomitant with increased expression of two anabolic markers COL II and aggrecan; these results were consistent with another study [42]. Disc degeneration occurs due to an imbalance between catabolism and anabolism in discs, leading to excessive ECM degradation. EVs can affect the ECM, including matrix degradation, matrix protein crosslinking, and matrix calcification [43]. Zhang et al. found that NPCs with activated autophagy release EVs and prevent ECM destruction [44].

In the following experiments, we studied the mechanism underlying the promoting effects of BMSC-EVs on NPC survival and inhibitory effects on the ECM degradation in NPCs. Our RNA sequencing results identified circ_0050205, as it exhibited the largest fold change in expression between IDD tissues and normal tissues. We found that circ_0050205 mediated the promoting effects of BMSC-EVs on NPC survival and inhibitory effects on the ECM degradation in NPCs. In contrast to this favorable circRNA, Song et al. identified an unfavorable circRNA, circ_0000253, that was carried by EVs derived from NPCs of different degenerative grades and whose expression was upregulated in degenerative NPCs [16]. Other favorable circRNAs that protect the intervertebral disc from degeneration were identified. For example, circARL15 expression was downregulated in IDD and its overexpression prevented NPC apoptosis but promoted NPC proliferation [45]. Knockdown of circSNHG5 expression in chondrocytes reduced cell proliferation, which was followed by degradation of ECM [46]. A circRNA-related ceRNA network has been studied in IDD. In the present study, we also demonstrated that circ_0050205 functioned as a ceRNA to sponge miR-665 and then upregulated GPX4 expression. We found an increased miR-665 expression in the NP tissues of IDD mice compared to sham-operated mice, which was consistent with other evidence [36]. It was reported that miR-665 silencing attenuated ischemia/reperfusion injury-induced ROS production and inhibited cardiomyocyte apoptosis during myocardial infarction [47]. GPX4 was first demonstrated to be a target gene of miR-665 in this study. The GPX4 is an antioxidant defense enzyme and belongs to the selenoprotein family, which could limit the activity of ferroptosis by reducing lipid peroxidation [48]. GPX4 knockdown in mice may contribute to the ferroptosis in kidney tubular cells, inducing acute renal failure, which was efficiently suppressed by Fer-1 and DFO [49]. In our study, GPX4 was poorly expressed in the NP tissues of IDD mice and the IDD cell model, revealing that NPC ferroptosis possibly results from the lack of GPX4 function. More importantly, when we overexpressed GPX4 in NPCs, inhibited ferroptosis concomitant with reduced ECM degradation was noted. Similarly, a recent study has also verified the involvement of GPX4 in OS-induced NPC ferroptosis in IDD [8]. Furthermore, the involvement of GPX4 in the circRNA-related ceRNA network has been revealed in previous studies. Previous data demonstrated that silencing of circFNDC3B limited GPX4 expression and enhanced ROS, total iron level, and Fe^2+^ in oral squamous cell carcinoma cells via the miR-520d-5p/SLC7A11 axis [50]. In addition, Xu et al. showed that circIL4R inhibited ferroptosis through the miR-541-3p/GPX4 axis [51]. For confirming the *in vitro* data, we surgically established IDD in mice and demonstrated the therapeutic effects of BMSC-EVs on IDD; additionally, we showed that the mechanism by which the circ_0050205/miR-665/GPX4 axis mediated the inhibitory effects of BMSC-EVs *in vitro* was also relevant for the effects BMSC-EVs *in vivo*.

## 5. Conclusions

**Figure 8:**
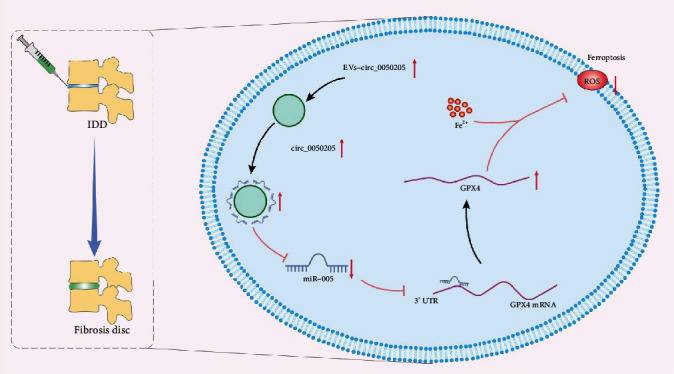
The mechanism graph of the regulatory network and function of circ_0050205. BMSC-EVs transferred circ_0050205 to NPCs where circ_0050205 acted as miR-665 sponge and upregulated the expression of the miR-665 target GPX4, thus accelerating NPC survival and reducing ECM degradation, ultimately preventing the IDD.

Overall, these experimental data revealed that circ_0050205 was transferred via EVs from BMSCs into NPCs. BMSC-EVs carrying circ_0050205 promote NPC survival and attenuate IDD progression by regulating miR-665 and GPX4 expression (Figure 8). These data reveal a unique regulatory network associated with NPC survival, namely, the circ_0050205/miR-665/GPX4 network. circ_0050205 might be an advantageous target for improving MSC-based cell strategies for treating IDD.

## Data Availability

The datasets generated and/or analyzed during the current study are available in the manuscript and supplementary materials.

## Supplementary Materials

Supplementary Materials Table S1: the primer sequences used for RT-qPCR. Supplementary Figure 1: effects of OS induction on NPC apoptosis and ECM degradation. Supplementary Figure 2: identification of the BMSCs. Supplementary Figure 3: results of bioinformatics analysis.
