# Supplementary material for: Bone Marrow Mesenchymal Stem Cell-Derived Extracellular Vesicles Carrying circ_0050205 Attenuate Intervertebral Disc Degeneration
Source: Oxid Med Cell Longev. 2022 Jul 5;2022:8983667. doi: 10.1155/2022/8983667 (PMC9277161; doi:10.1155/2022/8983667)
Supplement: Supplementary Materials — Table S1: the primer sequences used for RT-qPCR. Supplementary Figure 1: effects of OS induction on NPC apoptosis and ECM degradation. Supplementary Figure 2: identification of the BMSCs. Supplementary Figure 3: results of bioinformatics analysis. [file 8983667.f1.docx]

**Table S1** The primer sequences used for RT-qPCR.

| Gene | Sequence |
| --- | --- |
| circ_0050205 (mouse) | F: 5′-CATCTTCAACAGCGCCAACC-3′ |
|  | R: 5′-TGCCGATTCCTTTGGTCCAG-3′ |
| miR-665 (mouse) | F: 5′-AGGGGCCTCTGCCTCTATCCAGGATT-3′ |
|  | R: Reverse universal primer |
| U6 (mouse) | F: 5′-CGCACTTTACGGCTACCTCT-3′ |
|  | R: Reverse universal primer |
| GPX4 (mouse) | F: 5′-CGCCAAAGTCCTAGGAAACG-3′ |
|  | R: 5′-TATCGGGCATGCAGATCGAC-3′ |
| GAPDH (mouse) | F: 5′-CCCTTAAGAGGGATGCTGCC-3′ |
|  | R: 5′-ACTGTGCCGTTGAATTTGCC-3′ |

F: forward; R: reverse; RT-qPCR: reverse transcription quantitative polymerase chain reaction.

**
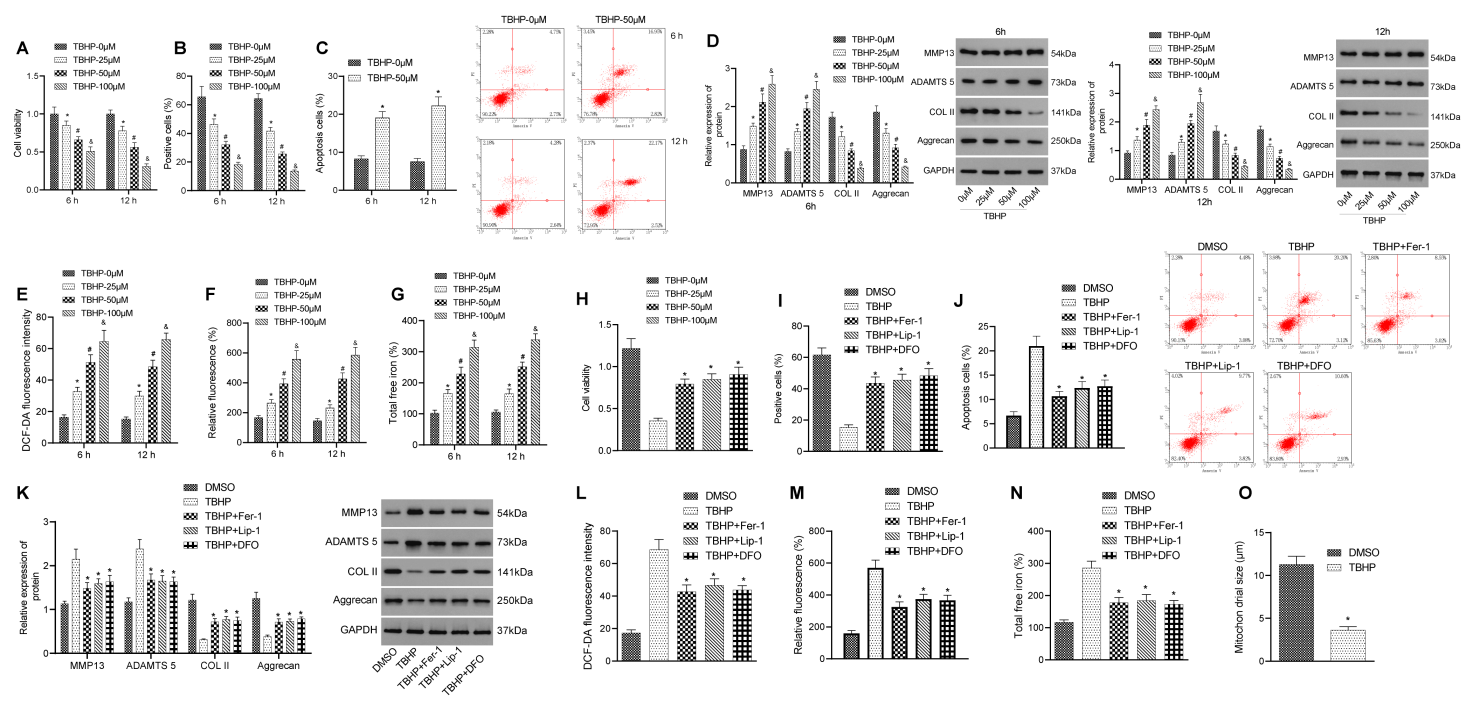
**

**Supplementary Figure 1** Effects of OS induction on NPC apoptosis and ECM degradation. a, CCK-8 assay for NPC viability following treatment with tBHP at varied doses for 6 h or 12 h. b, EdU staining for NPC proliferation following treatment with tBHP at varied doses for 6 h or 12 h. c, Flow cytometric analysis of NPC apoptosis following treatment with 50 μM tBHP for 6 h or 12 h. d, Western blotting of MMP13, ADAMTS 5, COL II, and Aggrecan proteins in NPCs following treatment with tBHP at varied doses for 6 h or 12 h. e, The ROS production was examined using DCF-DA kit in NPCs following treatment with tBHP at varied doses for 6 h or 12 h. f, Lipid peroxidation determined by C11-BODIPY581/591 in NPCs following treatment with tBHP at varied doses for 6 h or 12 h. g, The total free iron in NPCs following treatment with tBHP at varied doses for 6 h or 12 h. h, CCK-8 assay for NPC viability following treatment with tBHP and ferroptosis-specific inhibitors. i, EdU staining for NPC proliferation following treatment with tBHP and ferroptosis-specific inhibitors. j, Flow cytometric analysis for NPC apoptosis following treatment with tBHP and ferroptosis-specific inhibitors. k, Western blotting of MMP13, ADAMTS 5, COL II, and Aggrecan proteins in NPCs following treatment with tBHP and ferroptosis-specific inhibitors. l, The ROS production was examined using DCF-DA kit in NPCs following treatment with tBHP and ferroptosis-specific inhibitors. m, Lipid peroxidation determined by C11-BODIPY581/591 in NPCs following treatment with tBHP and ferroptosis-specific inhibitors. n, The total free iron in NPCs following treatment with tBHP and ferroptosis-specific inhibitors. o, TEM observation (upper: 5 μm; bottom: 2.5 μm) of mitochondrial damage and ferroptotic characteristics in NPCs following treatment with tBHP and ferroptosis-specific inhibitors, * *P* < 0.05 *vs*. DMSO. In panel A-C: * *P* < 0.05 *vs*. tBHP-0 μM, # *P* < 0.05 *vs*. tBHP-25 μM, & *P* < 0.05 *vs*. tBHP-50 μM. In panel H-N: * *P* < 0.05 *vs*. tBHP. Cell experiments were conducted three times independently.

**
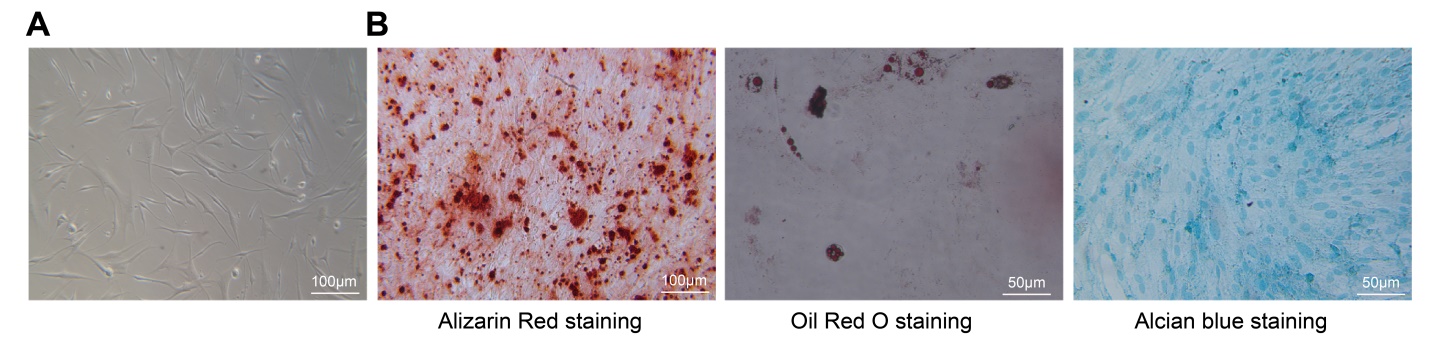
**

**Supplementary Figure 2** Identification of the BMSCs. A, Morphology of BMSCs observed under a light microscope (100 μm). B, Osteogenic, adipogenic and chondrogenic differentiation potential of BMSCs identified by Alizarin red standing (100 μm), oil red O staining (50 μm) and alcian blue staining (50 μm). Cell experiments were conducted three times independently.

**
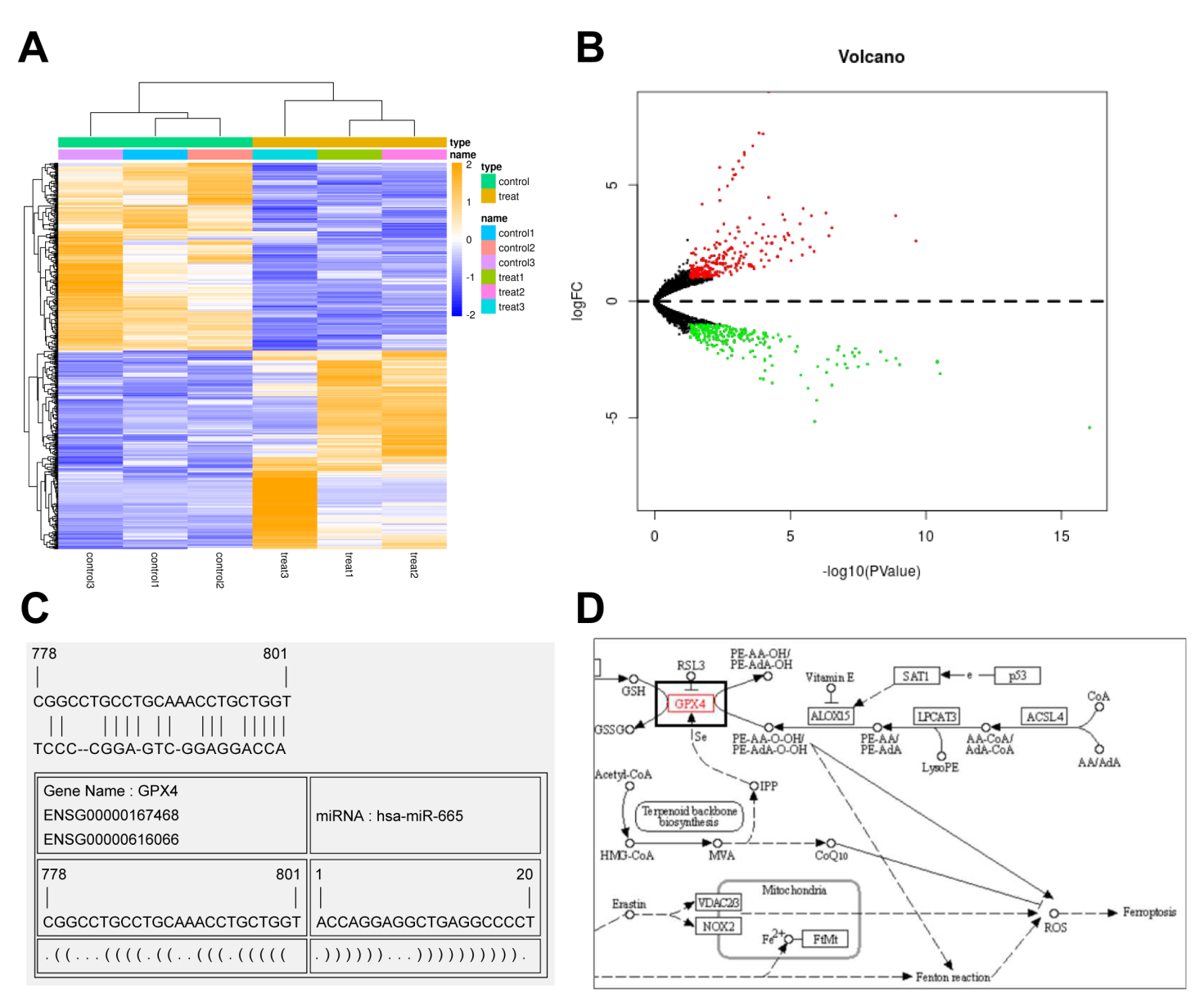
**

**Supplementary Figure 3** Results of bioinformatics analysis. a, A heat map showing differentially expressed circRNAs between NP tissues collected from IDD mice (n = 3) and NP tissues collected from normal mice (n = 3). b, A volcano map showing differentially expressed circRNAs between NP tissues collected from IDD mice (n = 3) and NP tissues collected from normal mice (n = 3). c, The miR-665 binding sites in the GPX4 3’UTR predicted by the RNA22 database. d, KEGG pathway enrichment analysis of GPX4.
